# Knowledge translation in Africa: are the structures in place?

**DOI:** 10.1186/s43058-020-00101-w

**Published:** 2020-12-11

**Authors:** James Avoka Asamani, Juliet Nabyonga-Orem

**Affiliations:** Inter-Country Support Team for Eastern & Southern Africa, Universal Health Coverage - Life Course Cluster, World Health Organization, P.O Box CY 348, 82-86 Enterprise/Glenara Roads, Highlands, Causeway, Harare, Zimbabwe

**Keywords:** Knowledge translation, Uptake of evidence, Decision-making, Policy development, Health sector

## Abstract

**Background:**

Contextualised evidence to generate local solutions on the progressive path to universal health coverage is essential. However, this evidence must be translated into action. Knowledge translation (KT) experts have highlighted the plausible mechanisms to foster the uptake of evidence. The objective of this study was to assess the extent to which structures are in place to boost uptake of evidence, in countries of the WHO African Region.

**Methods:**

Employing a cross-sectional survey, we collected data on the availability of structures to foster the uptake of evidence into policy in 35 out of the 47 member states of the WHO African Region. Data were analysed using a simple counting of the presence or absence of such structures.

**Results:**

Less than half of the countries had evidence collation and synthesis mechanisms. The lack of such mechanisms presents a missed opportunity to identify comprehensive solutions that can respond to health sector challenges. Close to 50% of the countries had KT platforms in place. However, the availability of these was in several forms, as an institution-based platform, as an annual event to disseminate evidence and as a series of conferences at the national level. In some countries, KT was mainstreamed into routine health sector performance review processes. Several challenges impacted the functionality of the KT platforms including inadequate funding and lack of dedicated personnel. Regarding dissemination of evidence, sharing reports, scientific publications and one-off presentations in meetings were the main approaches employed.

**Conclusion:**

The availability of KT platforms in the WHO African countries can be described as at best and non-existent at the worst. The current structures, where these exist, cannot adequately foster KT. Knowledge translation platforms need to be viewed as sector-wide platforms and mainstreamed in routine health sector performance reviews and policymaking processes. Funds for their functionality must be planned for as part of the health sector budget. Dissemination of evidence needs to be viewed differently to embrace the concept of “disseminate for impact”. Further, funding for dissemination activities needs to be planned for as part of the evidence generation plan.

## Introduction

The case has been made for contextualised evidence in the search for local solutions and innovation on the progressive path to universal health coverage (UHC). More pertinent to this aspiration is evidence being translated into action. Knowledge translation (KT) experts have been preoccupied with this topic for the last two decades and have highlighted plausible mechanisms to foster the use of evidence in decision-making. Amongst the favourable factors are the availability of high-quality, timely and contextualised evidence providing economically feasible recommendation; the Ministry of Health’s (MoH) capacity to lead the KT process; the availability of institutionalised platforms for engagement between researchers and policymakers including civil society; the mechanisms to coordinate evidence generation and synthesis mainstreamed within the MoH; and the partnerships for KT (involvement of stakeholders throughout the process to improve trust and build interest including communities) [1–3].

The type and quality of evidence have been a subject of debate with a preference for systematic reviews and peer-reviewed research [4]. In as much as this has worked well for biomedical policies, health systems and public health decisions call for additional evidence regarding affordability, political and community acceptability and implementation feasibility [1, 5]. Such evidence can be generated from systematic research, routine information systems, observation and community perceptions [6]. Evidence generated from the different perspectives on a given policy area needs to be synthesised to generate conclusions that can inform decision-making [7]. In ensuring this, evidence collation and synthesis mechanisms, as well as knowledge translation platforms (KTP), must be institutionalised in countries bringing together multiple disciplines and institutions. Evidence collation and synthesis mechanism are typically a policy-oriented process of triangulating from different pieces of evidence, for example, from routine information and monitoring systems, and research, which is pooled together to address specific policy questions—a mechanism often used in assessing health sector performance. A multidisciplinary team is usually put together to undertake the triangulation and synthesis of available evidence and produce a programme review report or health sector performance report. This mechanism is an essential pre-requisite for a functional KT platform which Kasonde and Campbell [8] defined as the medium of mutual exchange between researchers and policy actors, typically in the nature of a national- or state-level body which nurtures the linkages amongst the communities of researchers and policymakers to create cycles of policy-informed evidence generation and evidence-informed policy design and implementation.

In some African countries, partnerships for KT were institutionalised (in 2005) through Evidence-Informed Policy Networks (EVIPNet) which were established as knowledge translation platforms (KTP) to promote the systematic use of health research evidence in policymaking. These bring together policymakers, researchers and civil society to foster the uptake of the best scientific evidence in both policy development and policy implementation [9]. An evaluation conducted 7 years later showed promising results with reference to the instrumental use of evidence in programme design and policymaking, policymakers’ recognition of the role of evidence and specifically asking researchers to generate evidence on topical issues [10]. However, weaknesses in dissemination were highlighted as a gap. Poor dissemination of evidence has also been highlighted by other scholars who emphasise the importance of diversified mechanisms of dissemination, mapping of the target audience and tailoring messages, the media as an effective ally and the use of simple language [11, 12].

The definition of KT indeed embodies these favourable parameters as “the dynamic, iterative process including the synthesis (… emphasising the need for synthesis platforms..), dissemination, exchange (… the need for effective dissemination as a two-way mechanism..) and ethically sound application of knowledge to improve health, strengthen the health care system and provide more effective health services and products (…. the need for broad evidence beyond the efficacy of interventions…)” [13]. In this article, we use KT and uptake of evidence interchangeably.

The objective of this study was to assess the extent to which structures are in place to foster the uptake of evidence which is an essential element in the progressive realisation of UHC, in countries of the WHO African Region. Much of the work in the area of KT has focussed on defining what KT is and what it takes to translate evidence into policy with reference to a specific policy process which may not necessarily require all the KT structures to be in place. Attempts to detail required structures for KT have derived mainly from published literature which focused largely on high-income countries. Our analysis contributes to available literature from two fronts, namely, assessing the extent to which there is the presence of a broad range of structures which are beneficial to a wide range of policy process and, secondly, employing primary data from a large set of African countries.

## Methods

As part of a broader assessment in which data collection was conducted between December 2017 and August 2018, we employed a cross-sectional survey design using a semi-structured mailed questionnaire. All the 47 member states of the WHO Africa Region were included in the survey, and 35 responded (response rate of 74%). Prior to undertaking the study, country teams (MoH research focal person/designate, head of the national health research institute and a researcher from one of the research institutes that were selected by the MoH) were trained on filling in the questionnaire. The Ministry of Health focal point for research or the head of the national health research coordination institute primarily completed the questionnaire as the arrangements differed from one country to another. The completed questionnaire was validated by an in-country team comprising representatives of institutions conducting health research, the head of the national research coordination institution, the focal point for research in the WHO Country Office and the focal point for research in the Ministry of Health. Data were collected on the structures that foster knowledge translation specifically the availability of (1) an evidence collation mechanism; (2) platform for translating and communicating research to inform health policy and practice, as well as the performance of the platform and challenges faced; and (3) ways in which evidence is disseminated.

### Data analysis

Data were processed in a Microsoft Excel® spreadsheet and basic descriptive and comparative analysis conducted.

### Ethical clearance

This study was a standing request by the Ministers of Health of the WHO African Region, and the ethical approval to undertake the survey was granted by the WHO African Regional Office’s Ethics Review Committee.

## Results

### Availability of an evidence collation and synthesis mechanism

A lot of evidence is generated in countries through research, routine information systems and evaluation studies. Further, the evidence is generated on different aspects ranging from health systems, biomedical science and service delivery. No single study can provide all required answers to inform policy decisions, thus the need for collation and synthesis of all available evidence.

As shown in Table 1, evidence collation mechanisms were only available in 15 countries (43% of countries). In Kenya, such a mechanism was mainstreamed within the National Health Observatory. The lack of such mechanisms presents a missed opportunity to identify possible comprehensive solutions that can respond to health sector challenges.

**Table 1:** Availability of evidence collation and synthesis mechanisms and KT platforms

	Countries
Availability of an evidence collation and synthesis mechanism (*N* = 35)	
Yes (15 countries)	Tanzania, Cameroon, Ethiopia, Gabon, Kenya, Guinea-Bissau, Liberia, Malawi, Mali, Senegal, Niger, Rwanda, Ghana, Zambia, Zimbabwe
No (20 countries)	Gambia, Lesotho, Seychelles, Nigeria, Eswatini, Botswana, Namibia, Congo, Cote D’Ivoire, Benin, Burundi, Mauritania, Mauritius, Democratic Republic of Congo, Carbo Verde, Mozambique, Eritrea, Sierra Leone, South Sudan, Uganda
Availability of platform for translating and communicating research (*N* = 35)	
Yes (20 countries)	Gambia, Tanzania, Cameroon, Ethiopia, Eswatini, Kenya, Guinea-Bissau, Lesotho, Liberia, Malawi, Mali, Mozambique, Seychelles, Senegal, Niger, Rwanda, Ghana, Zambia, Uganda, Zimbabwe
No (15countries)	Gabon, Nigeria, Botswana, Namibia, Congo, Cote D’Ivoire, Benin, Burundi, Mauritania, Mauritius, Democratic Republic of Congo, Carbo Verde, Eritrea, Sierra Leone, South Sudan

### Availability of platform for translating and communicating research

Sixteen out of 35 countries (46%) had KT platforms in place. However, the availability of these was in several forms, as an institution-based platform organised and managed by the national research institute (Cameroon, Ethiopia, Tanzania), as an annual event to disseminate evidence (National Health Research Forum—Lesotho and The Gambia; National Health Conference—Liberia) and as a series of conferences at the national level (Eswatini, Zimbabwe, Tanzania). In some countries such as Rwanda, knowledge translation was mainstreamed into routine health sector performance review processes. In Kenya and Guinea-Bissau, although reported as “in place”, the platform was still under development to be embedded in the National Health Observatory (Kenya) and as part of the committee to implement the national research agenda (Guinea-Bissau).

Noteworthy is the innovative approach employed by Senegal that instituted a capacity building programme on knowledge translation for policymakers and researchers. Niger, on the other hand, has an institutionalised mechanism for multi-sectoral consultation and exchange in health research.

Several challenges impacting the functionality of the KT platforms were reported. Inadequate funding was a major issue, and several annual dissemination forums had not been held in several countries, for example, The Gambia that had not held such forums for 4 years due to lack of funding. The poor synthesis and presentation of research results to guide policy dialogue were also noted as a hindrance (Tanzania).

Suboptimal functionality was reported due to the lack of dedicated personnel. These challenges are exacerbated by not clearly defining the role of institutions (research institutions, media and leadership roles), weak capacity of researchers and policymakers and the environment-related issues such as Information and Communication Technologies (ICT) to ensure the functionality of KT platforms. There seem to be no agreed set of indicators for measuring the functionality of KT platforms, and using proxy indicators to assess progress made on the challenges described above will be a good starting point worth a future study.

### Dissemination of evidence

As shown in Table 2, countries employ several approaches to disseminate evidence, the commonest being oral communication through meetings followed by scientific publications. The use of the media is minimal (only five countries) despite being influential. The use of virtual libraries in Sierra Leone and Mauritius is not a common occurrence in Africa, and ease of access to such by stakeholders needs to be explored.

**Table 2:** Dissemination mechanisms employed by countries

Mechanism	Country
Communication (oral, displays); presentation in meetings, conferences, symposiums, and seminars (national and international) (24 countries)	Democratic Republic of Congo, Benin, The Gambia, Tanzania, Cameroon, Ethiopia, Kenya, Guinea-Bissau, Liberia, Malawi, Mali, Nigeria, Benin, Mauritania, Senegal, Niger, Carbo Verde, Mozambique, Rwanda, Sierra Leone, Ghana, Zambia, Zimbabwe, Mauritius
Television broadcasts, radio, press releases (5 countries)	Democratic Republic of Congo, Guinea-Bissau, Nigeria, Rwanda, Zimbabwe
Scientific publications (18 countries)	Democratic Republic of Congo, Benin, Tanzania, Cameroon, Ethiopia, Kenya, Guinea-Bissau, Liberia, Nigeria, Benin, Mauritania, Senegal, Niger, Rwanda, Sierra Leone, Ghana, Zambia, Mauritius
Sensitisation campaigns (1 country)	Cameroon
Posting on websites (8 countries)	Liberia, Nigeria, Niger, Carbo Verde, Sierra Leone, Ghana, Zambia, Mauritius
Sharing reports by email (15 countries)	Tanzania, Cameroon, Ethiopia, Kenya, Guinea-Bissau, Liberia, Nigeria, Benin, Mauritania, Senegal, Niger, Rwanda, Sierra Leone, Ghana, Zambia
Annual reports, bulletins, newsletters, posters, leaflets, flyers, magazines, policy briefs (7 countries)	Nigeria, Senegal, Democratic Republic of Congo, Carbo Verde, Ghana, Zambia, Zimbabwe
Virtual library (2 countries)	Sierra Leone, Mauritius

## Discussion

Nearly half of the countries had evidence collation and synthesis mechanisms and knowledge translation platforms to foster the uptake of evidence in policy development and implementation. No single source of evidence can provide comprehensive options to a policy question, and as such evidence from routine systems, several research studies and evaluation reports must be synthesised to provide timely and comprehensive answers. Knowledge translation platforms have been instrumental in improving the uptake of evidence [14]. El-Jardali et al. [14] emphasise that these must be integrated and institutionalised within the policymaking processes. This implies that they must be part of the health sector structures bringing together policy actors and researchers to facilitate synthesis, interpretation and uptake of evidence on the one hand and, on the other hand, the policy questions then informing future research (policy-informed evidence generation). Rwanda presents a best practice that can be emulated by other countries where knowledge translation was mainstreamed into routine health sector performance review.

The absence of such presents a missed opportunity to realising evidence-based dialogue and decision-making on one hand and waste of resources on the other. Generation of evidence is not an end in itself, and as such, its use in improving service delivery needs to be viewed as a return on investment. El-Jardali et al. [14] highlight the role of KT platforms in the COVID-19 response as important platforms in providing timely evidence to guard against misinformation and political interest and bridge the science and policy and implementation gap.

Where KT platforms existed, there was an inherent weakness that may impact negatively on their functionality—for example, being institution-based limits the extent to which stakeholders can be involved because of the limited convening power of national research institutes. Being annual events does not offer much benefit, given the fact that decision-making is an ongoing process. Worst still, several of these annual events had been missed due to the lack of funding. The poor synthesis and presentation of research results, as was reported in Tanzania, negatively impacts the uptake of evidence. This situation reinforces the need to strengthen individual and institutional capacities in not just research but also policy-oriented evidence synthesis and science communication.

Amongst the barriers to uptake of evidence is the lack of appreciation of each other’s world of operation between policymakers and researchers [15]. To this end, we note the initiative undertaken by Senegal to address this gap through an institutionalised capacity building programme on KT, which could be an incentive for researchers and policymakers to work together.

Regarding dissemination of evidence, the approaches employed predominantly were sharing reports, scientific publications and presentation in meetings. Such approaches do not offer much reach for several reasons; access to scientific publications is a challenge because of limited access to the Internet and journal that are not open access. This is compounded by the poor reading culture, which is a documented barrier [16]. The limited use of the media presents a missed opportunity given their capacity to mobilise communities to demand policy evolution [12]. There was no mention of the use of knowledge brokers and personal communication which have proven to be effective [3, 17]. Scholars emphasise the importance of effective dissemination of evidence making reference to simplified messages tailored to different audiences and employing multiple approaches [2, 3]. Amongst noted effective strategies are demand-driven approaches (where researchers respond to policy-makers demand for evidence), and these have been employed in some settings with success.

Although KT structures are still suboptimal, there is documented evidence of the improved quality of health policies underpinned by concrete context-appropriate evidence in Zambia, Uganda, Cameroon, and Ethiopia where KT platforms played an integral role [8]. In these success stories, the contribution of development partners in establishing evidence and collation synthesis mechanisms has been immense. In the case of Zambia and Senegal, high-level interest and support from the government have ensured sustainability beyond the start-up support from development partners.

### Study limitations

Our assessment focused on the extent to which structures are in place to foster the uptake of evidence in countries of the WHO African Region. We did not undertake an in-depth assessment of the functionality of these structures and possible solutions to address identified challenges. We, however, believe our findings provide valuable information that can spur action in improving the uptake of evidence. In as much as the functionality of such structures is important, these must be in place in the first place.

## Conclusion

The availability of KT platforms in the WHO African countries can be described as suboptimal at best and non-existent at the worst. The current structures, where these exist can not adequately foster KT. The starting point would be to have structures established where these are lacking. Further, knowledge translation platforms need to be viewed as sector-wide platforms and mainstreamed in routing health sector performance review and policymaking processes. Funds for their functionality must be planned for as part of the health sector budget. Regarding the dissemination of evidence, sharing reports, scientific publications and one-off presentations in meeting approaches employed currently in the majority of countries have already been shown to have minimal impact. Dissemination of evidence needs to be viewed differently to embrace the concept of “disseminate for impact”. Further, funding for dissemination activities needs to be planned for as part of the evidence generation plan.

## Data Availability

The data set generated and analysed during the current study is available with the WHO Africa Region Office and the European and Developing Clinical Trials Partnership.

## Abbreviations

KT: Knowledge translation
MoH: Ministry of Health
WHO: World Health Organization
